# Nanoscale Structure and Spectroscopic Probing of Aβ1-40 Fibril Bundle Formation

**DOI:** 10.3389/fchem.2016.00044

**Published:** 2016-11-22

**Authors:** Katarzyna M. Psonka-Antonczyk, Per Hammarström, Leif B. G. Johansson, Mikael Lindgren, Bjørn T. Stokke, K. Peter R. Nilsson, Sofie Nyström

**Affiliations:** ^1^Department of Physics, Norwegian University of Science and Technology NTNUTrondheim, Norway; ^2^IFM-Department of Chemistry, Linköping UniversityLinköping, Sweden

**Keywords:** amyloids, amyloid formation, AFM, hyperspectral imaging, SPR, fibrillation, oligothiophenes

## Abstract

Amyloid plaques composed of fibrillar Amyloid-β (Aβ) are hallmarks of Alzheimer's disease. However, Aβ fibrils are morphologically heterogeneous. Conformation sensitive luminescent conjugated oligothiophenes (LCOs) are versatile tools for monitoring such fibril polymorphism *in vivo* and *in vitro*. Biophysical methods applied on *in vitro* generated Aβ fibrils, stained with LCOs with different binding and fluorescence properties, can be used to characterize the Aβ fibrillation in depth, far beyond that possible for *in vivo* generated amyloid plaques. In this study, *in vitro* fibrillation of the Aβ1-40 peptide was monitored by time-lapse transmission electron microscopy, LCO fluorescence, and atomic force microscopy. Differences in the LCO binding in combination with nanoscale imaging revealed that spectral variation correlated with fibrils transforming from solitary filaments (Ø~2.5 nm) into higher order bundled structures (Ø~5 nm). These detailed *in vitro* experiments can be used to derive data that reflects the heterogeneity of *in vivo* generated Aβ plaques observed by LCO fluorescence. Our work provides new structural basis for targeted drug design and molecular probe development for amyloid imaging.

## Introduction

A polypeptide chain has the ability to fold into a functional and unique 3D structure depending on the intrinsic properties of its amino acid sequence, the surrounding environment which *in vivo* include the cellular quality control machinery, such as molecular chaperones (like Hsp70, PPIase; Sörgjerd et al., 2006; Moparthi et al., 2010), and other proteins and enzymes (Gregersen et al., 2006; Chen et al., 2008; Dill and MacCallum, 2012). Partially folded or misfolded proteins and peptides that escape the quality control machinery and evade degradation have the tendency to form insoluble clusters that are prone to aggregate into larger ordered structures called amyloid fibrils (Dobson, 2003). Proteins and peptides that are misfolded i.e., are not in their functional conformation, lack the normal biological activity and may attain properties detrimental to the cells and tissues harboring them. There is a significant number of severe human pathologies that are identified and diagnosed based on extracellular deposition of insoluble protein and peptide aggregates in the form of amyloid fibrils (Stefani and Dobson, 2003; Chiti and Dobson, 2006; Harrison et al., 2007). These diseases include neurodegenerative conditions like Alzheimer's, Huntington's, Parkinson's, Creutzfeldt-Jakob, and other prion disorders, but also systemic diseases such as systemic amyloidosis. Although, the fibrils were originally recognized in connection with clinical disorders, it becomes increasingly apparent that the folding to misfolded states can be deliberately achieved *in vitro* by many different polypeptides when exposed to appropriately chosen solution conditions (low pH, lack of specific ligands, high temperature, and presence of specific salts, or co-solvents). This fact provides a strong support to the hypothesis that the ability to form fibrils can be considered as inherent and generic characteristics of the polypeptide chain emerging from the physical and chemical properties of the polypeptide chain itself (Dobson, 2006). Moreover, there is growing evidence that in nature there is a wide variety of organisms that are able to take advantage of the controlled aggregation of specific proteins and peptides to generate fully functional structures for beneficial reasons (Fowler et al., 2007; Otzen, 2010).

Various proteins and peptides that have been demonstrated to misfold into amyloids do not share similarities in size, amino acid composition, sequence, and structure. Notwithstanding they can be converted into amyloid fibrils that share a series of distinct tinctorial (affinity for Congo red with concomitant green birefringence) and biophysical characteristics both in their overall morphology and in their internal structure. The amyloid fibrils show very characteristic appearance in EM and AFM images as long, linear, composed of 1-6 protofilaments, unbranched fibers with few nanometers thickness, frequently with repeating twist at regular intervals, but with variable pitch for individual fibril types (Adamcik et al., 2010; Usov et al., 2013). They share a common core structure being composed of a network of densely packed hydrogen bonds that stabilize an elongated assembly of β-strand structural motifs perpendicular to the fibril axis and with 0.48 nm separation.

**Figure 1:**
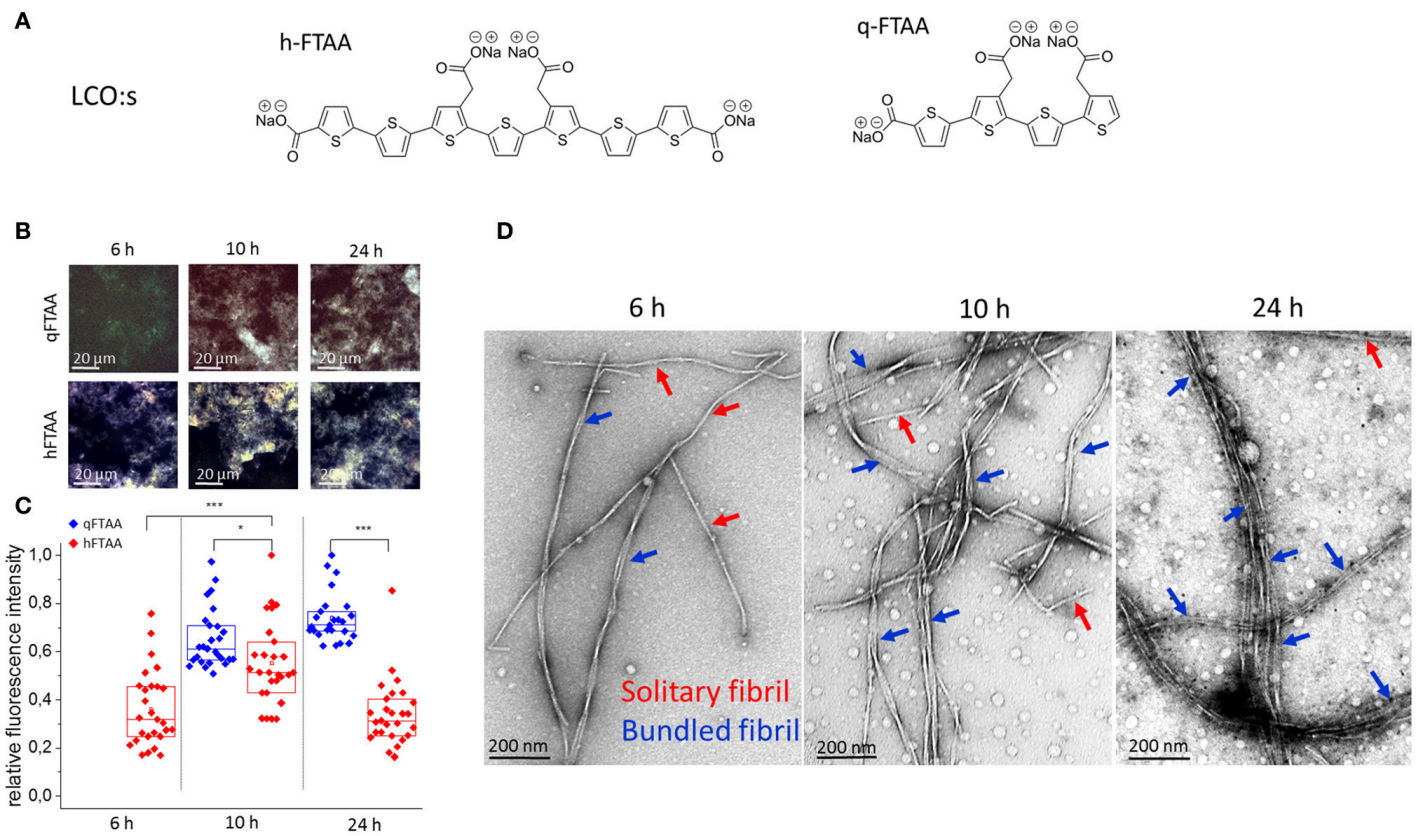
**(A)** Chemical structure of the in-house synthesized LCOs used. **(B–D)** Samples from selected time points during fibrillation were stained with q-FTAA and h-FTAA for hyperspectral imaging as well as prepared for TEM analysis **(B)** Representative images taken with identical microscope settings within the dynamic range of the detector, using hyperspectral imaging with long pass filter in epifluorescence mode. **(C)** Relative maximum fluorescence intensities from the entire images. Significance was calculated using two-sample *t*-test, ^*^p < 0.05, ^***^p < 0.001. **(D)** Representative TEM images from selected time points. At 6 h the q-FTAA fluorescence was too weak to give reliable fluorescence data (**B**, top left and **C**, data missing). The relative amount of multi-filamentous bundled fibrils as demonstrated by TEM was lower at 6 h as compared to 10 and 24 h. q-FTAA fluorescence increases relative to h-FTAA fluorescence **(C)** as the presence of fibril bundles becomes more pronounced **(D)**. Spherical globular species likely oligomers were observed at all time-points.

One of the most extensively characterized amyloid fibril protein is the major constituent of the amyloid deposits (neuritic plaques in the gray matter of the brain) that is one of the main hallmarks of Alzheimer's disease, namely amyloid beta peptide (Aβ). The precursor of Aβ peptide is a transmembrane protein APP (amyloid precursor protein) that is concentrated in the synapses of neurons. The Aβ peptide is derived as a result of proteolytic cleavage of APP by β- and γ-secretase that cleaves APP in different steps (Nunan and Small, 2000). Depending on the exact point of cleavage by γ-secretase, two main forms of Aβ, composed of 40, and 42 amino acid residues, respectively, are produced. The formation of Aβ plaques *in vivo* has been well studied over the past decades, using transgenic mice (mouse models reviewed in Puzzo et al., 2015). We have previously used both *in vitro* derived Aβ amyloid fibril from recombinant sources and *in vivo* derived Aβ amyloid in the brains of transgenic mice to elucidate the process of amyloid rearrangement over time (Nyström et al., 2013). By monitoring both *in vitro* and *in vivo* derived Aβ amyloid from a number of different time points using oligothiophenes, conformation sensitive fluorescent probes (Nyström et al., 2013), we were able to observe a maturation process taking place over time. The heptameric oligothiophene h-FTAA (Figure 1A) displays red fluorescence with an emission peak at a wavelength λ = 540 nm when bound to amyloid fibrils as well as pre-amyloid (that is, non-thioflavinic or congophilic) species. In contrast, blue-shifted fluorescence with emission maximum at λ = 500 nm from the tetrameric oligothiophene, q-FTAA (Figure 1A), was only observed from mature amyloid fibril structures similar to those detected by Thioflavin T and Congo Red. The time scale observed for maturation of *in vitro* fibrillation of recombinant Aβ is 24 h and for *in vivo* derived Aβ amyloid in transgenic mice it is 18–30 months. The variation was more robust for Aβ1-40 and the alterations within plaque cores were more prominent in APP23 mice expressing high levels of Aβ1-40 (Sturchler-Pierrat et al., 1997) than in APP/PS1 mice expressing higher levels of Aβ1-42 compared to Aβ1-40 (Radde et al., 2006).

In the current work we study Aβ1-40 fibrils to unravel the nanometer scale differences between early, immature fibrils, and the later mature, and morphologically different fibrils. By combining atomic force microscopy (AFM), transmission electron microscopy (TEM), hyperspectral imaging (HSI) of q-FTAA, and h-FTAA emission and surface plasmon resonance (SPR) we were able to assign different stages on the amyloid formation and maturation pathway as solitary fibrils being formed initially that over time further matured into multi-filamentous bundles. These findings allowed us to hypothesize on the structural composition of polymorphisms found in senile plaque *in vivo* and structures recognized by the respective LCO.

## Materials and methods

### Fibril preparation

Aβ1-40 peptide lyophilized from HFIP was purchased from R-Peptide (Bogart, USA), resuspended to 1 mg/ml in 2 mM NaOH and stored at −20°C until needed. At time of fibrillation, the 1 mg/ml stock was thawed and PBS (0.14 M NaCl, 0.0027 M KCl, 0.01 M ${\text{PO}}_{4}^{3-}$, pH 7.4, Medicago, Uppsala, Sweden) was added to give a final Aβ concentration of 10 μM. Bulk fibrillation reactions were set up in 15 ml polystyrene tubes at 37°C without shaking. Sample tubes were vortexed prior to each time point sampling. Unseparated samples were stained and analyzed as described in the sections below. For isolation and analysis of soluble material, the supernatant after centrifugation at 70,000 g for 18 h, at 4°C was collected. Samples for time scan imaging were put on ice at the respective time point to prevent further fibrillation. In solution fluorescence of unseparated samples and supernatants was measured using a Tecan Saphire II or Tecan M1000 fluorescence plate reader (Tecan, Austria) with an excitation wavelength of 440 nm.

### *Ex situ* staining and fluorescence imaging of Aβ1-40 fibrils

Samples from selected time points, both unseparated and supernatants, were extracted from the bulk fibrillation and LCO was added to reach final concentrations 13 nM q-FTAA and 7 nM h-FTAA, respectively. The samples were allowed to sediment by gravity overnight. Droplets of 3 μl were placed on superfrost ultra plus microscopy glass and allowed to dry at ambient conditions. The samples were mounted with Dako mounting medium (Dako, Glostrup, Denmark) and analyzed the following day. Two representative images from each sample were collected and 27 regions of interest (essentially covering the entire visible image) from each image were used for calculation of the relative fluorescence intensities at 500 and 540 nm, respectively.

### TEM

Samples collected at 6, 10, and 24 h were analyzed by TEM. Both unseparated samples and the supernatants collected after ultracentrifugation at 70,000 G for 18 h at 4°C were applied to carbon-B coated copper mesh grids (Ted Pella Inc.), allowed to settle, washed with MQ-water and stained with 2% uranyl acetate prior to drying at ambient conditions. Images were collected using a Jeol JEM 1230 microscope set to 100 kV equipped with a CCD camera (Gatan).

### SPR

Surface plasmon resonance data were recorded using a Biacore 2000 instrument (GE Healthcare, Uppsala, Sweden) at 25°C. PBS, pH 7.4, was used as a running buffer. A CM5 chip was functionalized using conventional amine coupling chemistry as previously described (Johansson et al., 2015). Oligothiophenes q-FTAA-azide and h-FTAA-azide (150 μM, 50 μL) were injected at a flow rate of 5 μL/min, in separate flow channels, two q-FTAA and two h-FTAA-azide channels. Aβ (1–40) fibrils (10 μM, 50 μL, in PBS), fibrillated at different time points and antibody 4G8 (1 μg/mL, 50 μL, in PBS), were injected at a flow rate of 20 μL/min. Sensograms were evaluated using BIAevaluation software version 4.1 (GE Healthcare). In total 24 samples were run (12 over each channel type). The sensograms allowed calculations of binding events during each injection (number of LCOs bound to the chip, number of Aβ molecules in respect to monomer and number of antibodies bound to each channel). From this result, the ratio between the number of Aβ monomers that were required for one antibody binding event was calculated.

### Dot blot

10 μM Aβ1-40 fibrils harvested after 48 h of fibrillation at 37°C in PBS were stained with q-FTAA or h-FTAA with a final LCO concentration of 200 nM and probe binding was confirmed by in solution measurement of fluorescence signature. The stained fibrils were serially diluted in several steps and 2 μl droplets from each stain and each dilution and were blotted onto pvdf membrane using a pipette, together with equivalent amounts of unstained fibril. 4G8 binding was performed over-night at room temperature followed by 30 min incubation with AP conjugated secondary antibody (RAM-AP, AbCam) and developed using and Immune star AP substrate (Biorad). Consecutive images were collected using an Image Quant LAS4000 system (GE healthcare) and analysis of intensity in each dot was performed using Image Quant TL (GE healthcare). Intensities for each dot were measured from four different exposures to accommodate variability due to exposure time.

### Histology

Animal handling was carried out in accordance with relevant guidelines of breeding and keeping transgenic animals as well as culling and collection of tissue for *ex vivo* experiments. Cryosections (10 μm) of cerebrum from an 18 months old APP23 mouse were rehydrated using consecutive ethanol and PBS dips and stained with the LCOs q-FTAA/h-FTAA as previously described (Nyström et al., 2013). 4G8 antibody (Covance, Princeton, NJ, USA) was diluted to 1 μg/ml in PBS and was left on the slide at 4°C overnight. The sections were washed and GAM647 secondary antibody (Thermo Scientific, Waltham, MA, USA) diluted 1:500 was allowed to incubate for 1 h on the tissue sample prior to washing and mounting with Dako fluorescence mounting medium (Dako, Glostrup, Denmark). Confocal images were collected with a Zeiss LSM 780 using the following settings; Excitation laser 458 nm, emission collected at 485–543 nm; excitation laser 488 nm, emission collected at 542–621 nm; excitation laser 640 nm, emission collected at 660–750 nm; objective 20 ×/0.8 M27.

### AFM

AFM imaging requires immobilization of the specimen to be imaged on a flat surface, commonly mica. To assure strong and effective binding of fibrillated material to mica the latter was modified accordingly. Freshly cleaved mica sheets were incubated in 1% (v/v) solution of trimethoxysilylpropyldiethylenetriamine (Sigma Aldrich, St. Louis, MO, USA) in 1 mM acetic acid for 30 min following the incubation in 0.5% (v/v) of glutaraldehyde (Sigma Aldrich, St. Louis, MO, USA) in MQ-water. After each step of incubation mica sheets were extensively rinsed with MQ-water and dried in a stream of compressed nitrogen. Such modified surfaces were used for binding the fibrillated material. A droplet of 12 μl of fibrillated Aβ1-40 peptide (diluted 1:3 in PBS, pH 7.4, immediately before deposition followed by thorough mixing) was placed onto modified mica and incubated for 15 min. The mica sheets were subsequently rinsed extensively with MQ-water to remove the PBS buffer and unbound fibrillated material. Rinsed mica sheets were dried in vacuum (ca. 0.1 mPa) to remove the leftovers of MQ-water and to enhance binding of fibrils. Dried mica sheets were stored in a dry cabinet till used for AFM imaging. Immobilized dry fibrillated Aβ1-40 peptides were imaged using Multimode AFM (Digital Instruments/Veeco) operated in tapping mode under ambient conditions. Silicon nitride cantilevers PPP-NCH (Nanosensors, nominal resonance frequency 204–497 kHz) were used. Overlapping of trace and retrace signal was used as a prerequisite for adequate and high-quality image acquisition.

## Results

### Aβ1-40 fibrillation monitored by LCO fluorescence and TEM

As the amyloid fibril formation can be studied with both fluorescent dyes and ultra-structural techniques, such as TEM and AFM, the fibrillation process of Aβ1-40 was simultaneously studied with two fluorescent oligothiophenes, q-FTAA and h-FTAA (Figure 1A) and with TEM (Figure 1D). To obtain a quantitative analysis of fibril morphologies in the context of LCO fluorescence we used TEM analysis of the specimens at time points where we detected distinct differences in LCO fluorescence signature. Aβ1-40 fibril preparations from time points corresponding to different phases of fibrillation (6, 10, and 24 h; Figure 1B) as deduced by q-FTAA/h-FTAA staining were isolated and imaged using TEM at various magnifications. Hyperspectral imaging of samples from different time points of Aβ1-40 fibrillation reactions revealed that the h-FTAA fluorescence was readily detected at all time points (Figures 1A,B).

On the other hand q-FTAA fluorescence was visually detectable albeit not of sufficient signal to record at 6 h (Figures 1B,C). The fluorescence intensity increased over time indicating a phase of morphologic alteration of the fibrils (Figures 1B,C). Samples from the same reaction and time points were also monitored by TEM (Figure 1D) to assign ultrastructural differences corresponding to the different fluorescence signatures. Importantly the increase in q-FTAA fluorescence and decrease of h-FTAA fluorescence correlated well with the increase of multi-filamentous fibrils over time. Thus, the fluorescence observed from q-FTAA and h-FTAA correlated to distinct morphotypes of Aβ1-40 aggregates that were observed by TEM.

**Figure 2:**
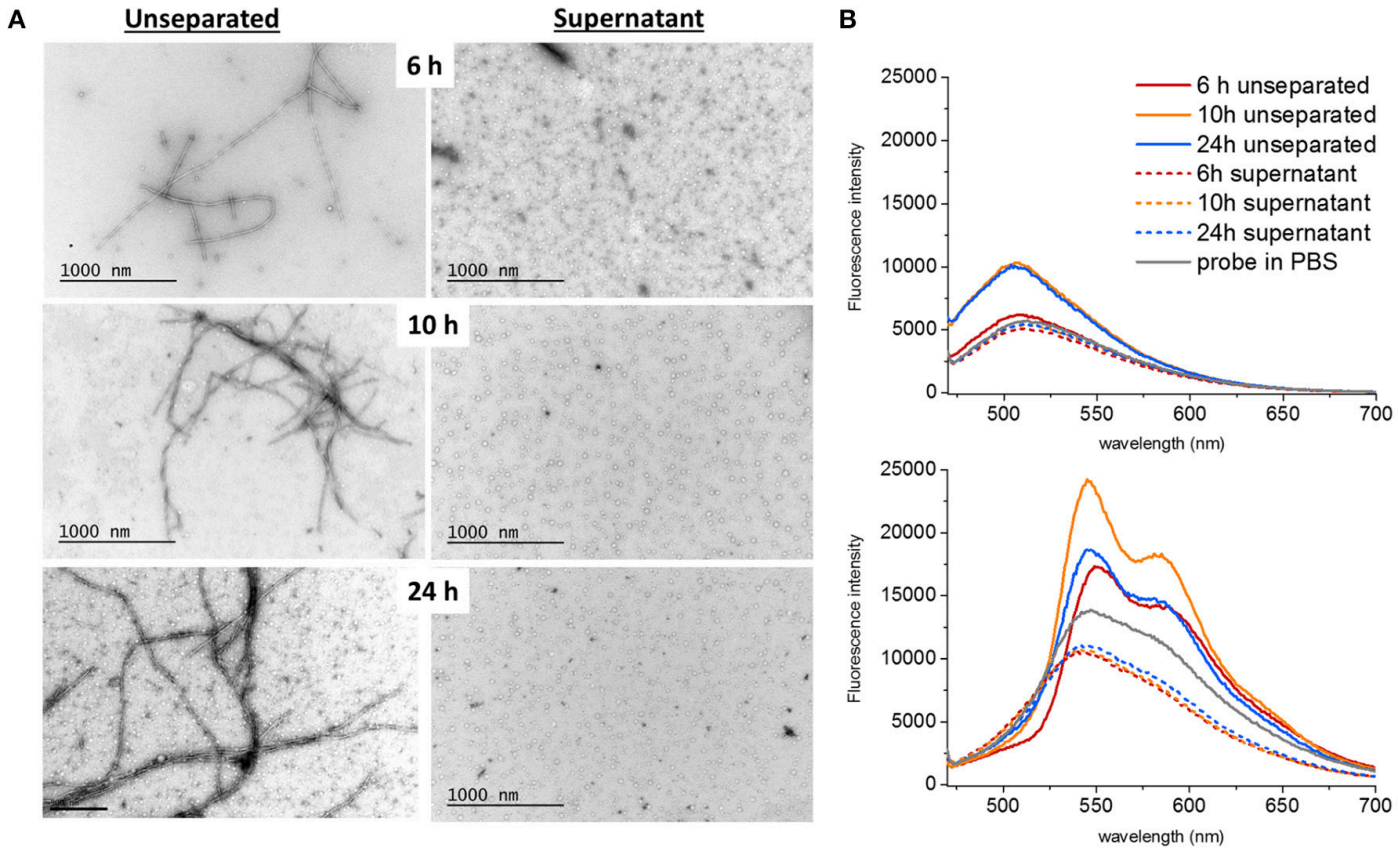
**Ultracentrifugation of different samples showed that both fibrils and oligomers were present at all time points. (A)** The supernatants only contained spherical oligomers and no fibrils. The proportion of bundled fibrils increased over time **(B)** q-FTAA (top panel) and h-FTAA (bottom panel) fluorescence measured in solution for both unseparated samples and supernatants showed that q-FTAA could not be detected at the early time point where no or very few bundled fibrils were detected by TEM. The supernatants were all devoid of increase in LCO fluorescence indicating that the spherical oligomers presented by TEM did not bind q-FTAA or h-FTAA in a fluorescent state.

To address the presence of soluble oligomers (Lee et al., 2011) at different time points of fibrillation, a separation step using ultracentrifugation at 70,000 g for 18 h at 4°C was performed. TEM images were obtained both of unseparated samples and of the supernatants after centrifugation (Figure 2A). The presence of LCOs as deduced by fluorescence intensity for both q-FTAA and h-FTAA diminished almost completely (>95%) from the supernatant (Figure 2B). It is important to note that the q-FTAA fluorescence spectrum is significantly increased as compared to free probe in PBS at 10 and 24 h but only very moderately at 6 h. The q-FTAA fluorescence in the supernatants was indistinguishable from the PBS control (Figure 2B, top panel). h-FTAA on the other hand displayed both intensity increase and spectral shift indicative of amyloid fibril binding already at 6 h in unseparated samples. Intriguingly, the h-FTAA fluorescence intensity was decreased in the supernatant as compared to free probe in PBS but with retained spectral profile (that of free probe), indicating that h-FTAA fluorescence might be quenched rather than enhanced by species present in the supernatant after centrifugation.

Spherical oligomers as well as fibrils were present at all the analyzed time points as deduced by TEM. Spherical oligomers were omnipresent in the supernatant and did not enhance the fluorescence of LCOs, rather the opposite showing quenching of h-FTAA, (Figure 2) indicating that these oligomers are not composed of sufficient regular cross-beta sheet structures to induce the fibril specific spectral signature of h-FTAA. Solitary single fibrils as well as bundled fibrils containing several adjacent and intertwined fibrils were deposited on the grids in the unseparated samples. Manual analysis of the TEM micrographs revealed that the portion of bundled multi-filamentous fibrils increased over time (Figures 1D, 2A, Table 1) while the length of individual fibrils remained rather constant. A fibril bundle factor (FBF) calculated as the total length of bundled fibrils divided by the total length of solitary fibrils shows that after 10 h the bundled fibrils dominate the fibrillation population. The fibril bundle fraction (FBf) calculated as the total length of bundled fibrils divided with the overall total length of fibrils reveals that bundled fibrils outnumbers the solitary fibrils by almost a factor 2 from 10 h fibrillation time and onwards. This data strongly supports that q-FTAA binding and formation of bundled fibrils are parallel events and indicates that bundled fibrils is a requirement for q-FTAA binding to Aβ1-40 fibrils.

**Table 1:** **Fibril bundle factor and fibril bundle fraction as function of fibrillation time**.

**Time (h)**	**n solitary fibrils**	**n bundled fibrils**	**Total length of solitary fibrils (nm)**	**Total length of bundled fibrils (nm)**	FBF^*^	FBf^**^
6	69	29	21,700	13,000	0.60	0.40
10	68	85	17,600	50,100	2.85	0.74
24	57	81	23,400	61,400	2.62	0.72

* $Fibril\;bundle\;factor\;(FBF)\;=\;\frac{Total\;length\;of\;bundled\;fibrils\;(nm)}{Total\;length\;of\;solitary\;fibrils\;(nm)}$

** $Fibril\;bundle\;fraction\;(FBf)\;=\;\frac{Total\;length\;of\;bundled\;fibrils\;(nm)}{\begin{array}{c} Total\;length\;of\;solitary\;fibrils\;(nm)\;+\\ Total\;length\;of\;bundled\;fibrils\;(nm) \end{array}}$

### Antibody epitope exposure in SPR

**Figure 3:**
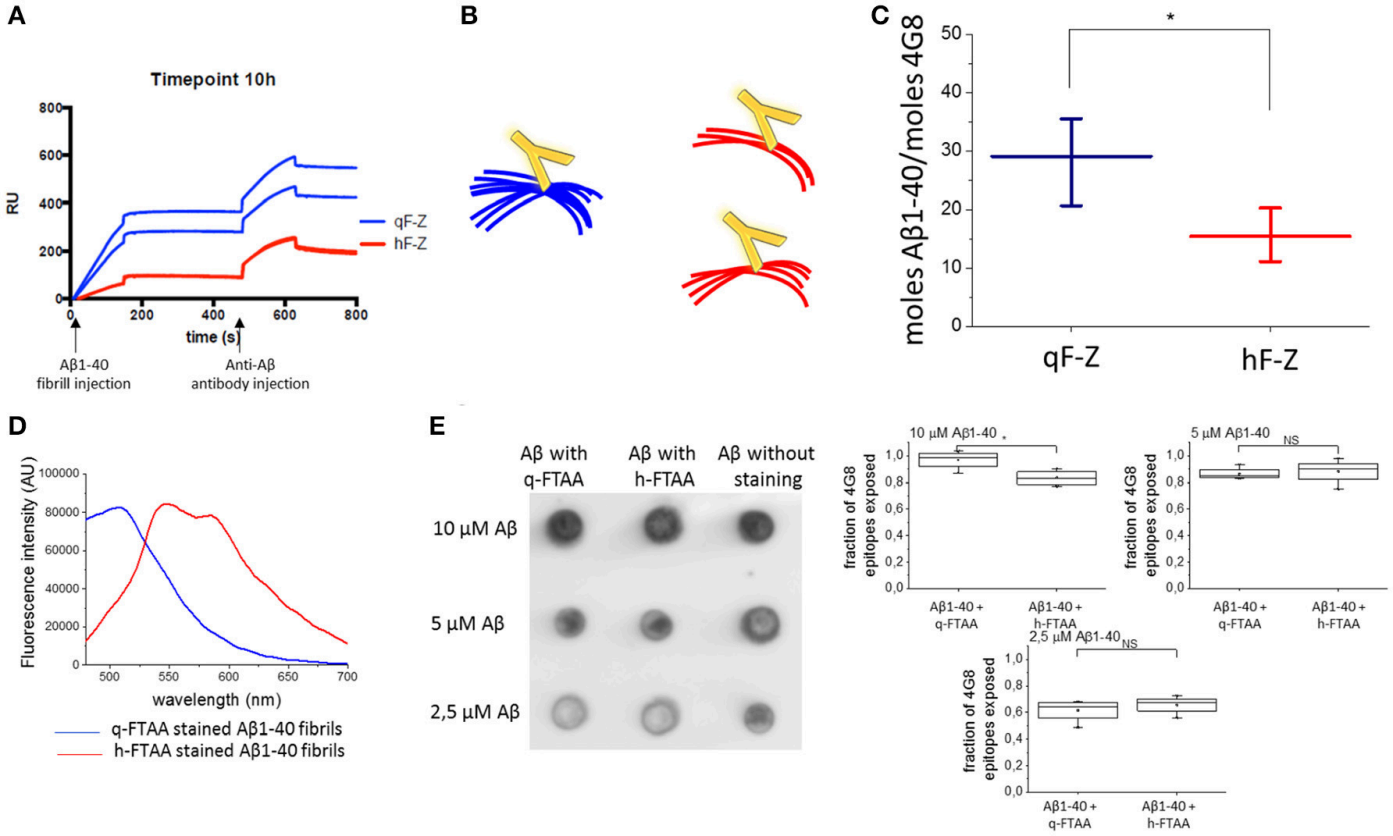
**Aβ antibody epitopes were less accessible when monitored by SPR. (A)** Shows sensograms from duplicate channels with LCO attached to the surface (with two sensograms for h-FTAA channels completely overlaying each other) and then Aβ fibril injection followed by anti-Aβ antibody injection. **(B)** Schematic representation of fibril bundling resulting in fewer exposed antibody epitopes. **(C)** Difference in number off Aβ monomers required for the binding of one antibody in the q-FTAA channel and h-FTAA channel, respectively. The mean of 12 measurements over each channel type with standard error of the mean is displayed. A two-sample *t*-test was used to show significant difference between the groups. **(D)** Fluorescence intensity for q-FTAA and h-FTAA respectively when bound to Aβ1-40 fibrils used for quantification of accessible 4G8 epitopes using dot blot. **(E)** Dot blot of LCO stained and unstained Aβ1-40 fibrils with 4G8 as primary antibody and quantification of relative signal intensity. **(D)** Fluorescence intensity displays equivalent binding of q-FTAA and h-FTAA. **(E)** The relative epitope exposure shows weak dependence on LCO binding. Significance was calculated using two sample *t*-test, ^*^p < 0.05.

To further investigate the differences in fibril structure between q-FTAA-binding and h-FTAA-binding fibrils, we employed a fluorescence independent SPR technique that discriminates between q-FTAA and h-FTAA binding morphotypes. In short, azide functionalized versions of q-FTAA and h-FTAA were attached to different channels on an SPR chip by a click chemistry protocol (two channels with each probe) and fibrillation material from different time points were injected over the modified chip as previously described (Johansson et al., 2015). The sensograms from the fibril injection and the subsequent anti-Aβ 4G8 antibody injection were used to calculate the relative difference between free antibody epitopes between the q-FTAA and the h-FTAA channels for each experiment (Figure 3A) based on the number of LCO molecules initially bound to the specific fibril.

The more bundled the fibrils are (q-FTAA binders) the fewer epitopes appear accessible for the antibody; hence more Aβ peptides with respect to monomer will be required for one 4G8 binding event (Figure 3B). The fibrils bound to the q-FTAA channels consequently displayed fewer 4G8 epitopes per q-FTAA bound fibril compared to h-FTAA which supports that more antibody epitopes were hidden in the interior of fibril bundles (Figure 3C).

As a control, a dot blot assay was performed to verify that the LCOs did not differently affect antibody binding properties due to variable 4G8 epitope competition. Fluorescence intensity measurements of q-FTAA and h-FTAA stained fibrils respectively demonstrate that both LCOs were bound to the fibrils (Figure 3D). Comparison of amount of epitopes exposed for 4G8 antibody binding reveals that equivalent epitope exposure is seen regardless of LCO but that the relative number of epitopes on stained compared to unstained fibrils decreases if the fibrils are diluted (Figure 3E).

### Antibody epitope exposure in histology

**Figure 4:**
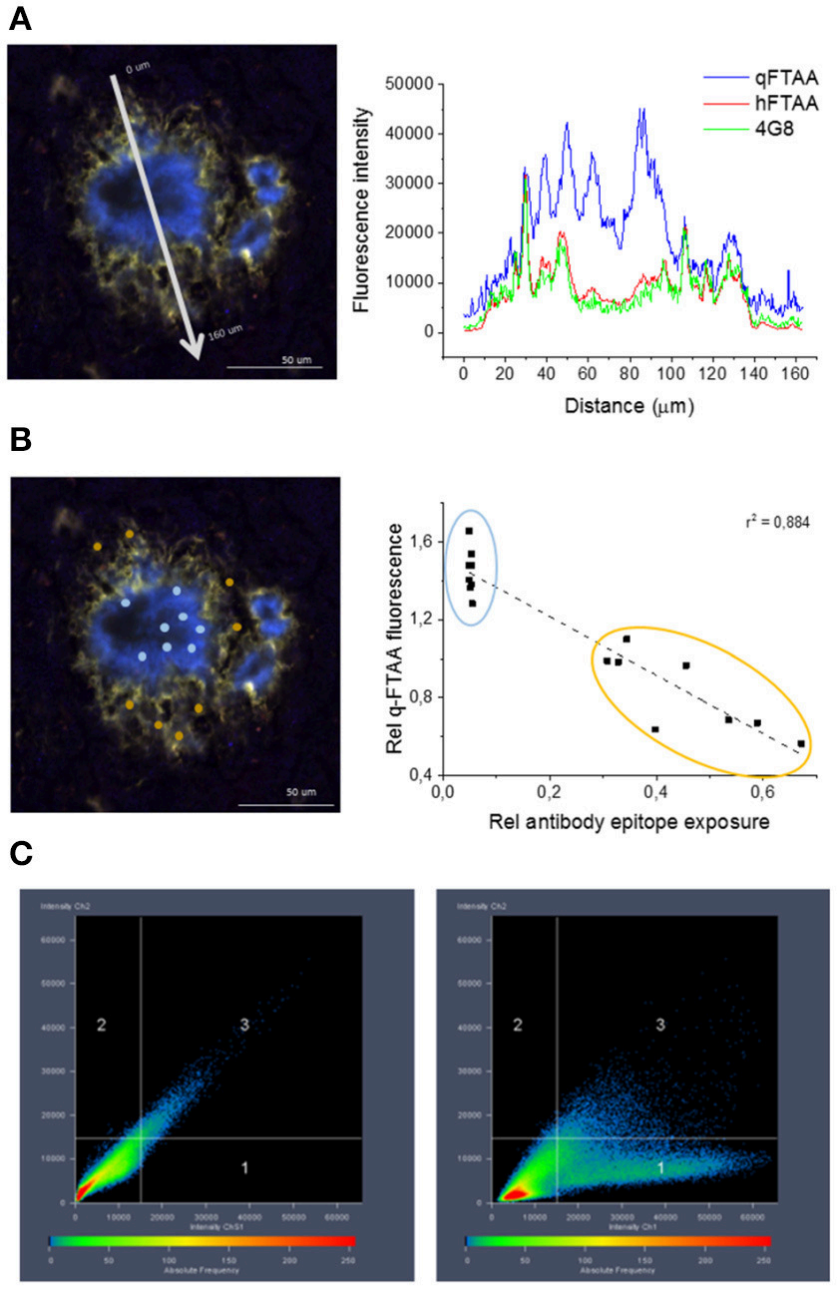
**Co-localization of 4G8 antibody, q-FTAA, and h-FTAA in histological sample of APP23 mouse. (A)** Intensities at different wavelengths over a cross section of one representative amyloid plaque in a 18 month old APP23 transgenic mouse brain. **(B)** Inverse correlation between signal from monoclonal 4G8-Aβ antibody and contribution of q-FTAA fluorescence (500 nm)/h-FTAA fluorescence (540 nm). ROIs selected for ratiometric analysis are indicated by colored dots and the corresponding data points are encircled with the same color in the graph. **(C)** High co-localization was displayed for h-FTAA and 4G8 antibody (left panel) and low for q-FTAA and 4G8 antibody (right panel).

Amyloid Aβ1-40 derived *in vivo*, e.g., in Aβ plaques in transgenic mice, display different stages of amyloid maturation over time (Nyström et al., 2013) similarly to that observed in the Aβ1-40 *in vitro* fibrillation assay. This was prominent in aged APP23 mice, which display high affinity for q-FTAA in the core of the plaques but no q-FTAA fluorescence in the plaque periphery. Therefore, we sought to understand if this maturation process is linked to the presence of bundled fibrils as observed over time for recombinant Aβ1-40 fibrils. Cryosections from 18 months old APP23 mouse brain were simultaneously stained with q-FTAA and h-FTAA, followed by 4G8 antibody detected with fluorescence at 640 nm. Confocal fluorescence imaging of the triple stained histology samples show that increased binding of q-FTAA toward the plaque core was accompanied by decreased density of fluorescence signal from the 4G8-anti-Aβ antibody (Figures 4A,B). The overall co-localization of 4G8 and h-FTAA was much higher than co-localization of 4G8 and q-FTAA (Figure 4C). These data implicates that antibody epitopes were less accessible in the fibril state where q-FTAA binds much more frequently compared to h-FTAA, supporting hidden 4G8 epitopes (Aβ residues 17–24) in mature putatively bundled fibrils *in vivo*. Taken together with the SPR experiments these data underline the biological relevance of bundled fibrils as an important parameter in amyloid maturation, as well as verifying that q-FTAA binding requires bundled fibrils.

### Time scan imaging with AFM

To further elucidate the mechanism of amyloid fibril formation and maturation over time, a high resolution monitoring of the fibrillation process was essential. Therefore, atomic force microscopy imaging was employed to examine Aβ1-40 at selected time points of the fibrillation. Fibrillated material collected at different time points was immobilized onto glutaraldehyde-modified mica and dried prior to AFM imaging.

**Figure 5:**
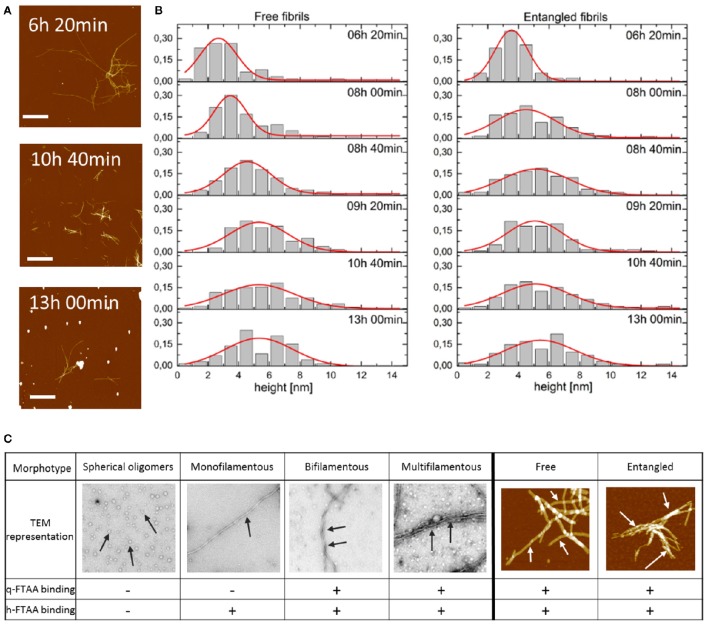
**(A)** AFM topographs presenting the fibrils in the process of Aβ1-40 fibrillation at the different time points. Scale bar represents 2 μm. Maximum height was chosen to be 20 nm to make the aggregated fibrils visible. **(B)** Distribution of the height of free and entangled fibrils of Aβ1-40 for consecutive time points of the fibrillation. Red lines represent Gaussian distribution fits to derive an average height of the fibrils. **(C)** Morphotypes of Aβ aggregates as depicted by TEM or AFM and indicated by arrows and their hypothesized binding propensity toward q-FTAA and h-FTAA, respectively.

Series of AFM images were collected for each time point and an overview of recorded images is presented in Figure 5A. Formed fibrils were straight and show no evidence of branching. Fibrils tended to cohere and associate with each other forming, as a result, rather big and spacious aggregates of entangled individual fibrils often with accompanying small globular structures, likely oligomers (Lee et al., 2011). Due to fibril association, there was only a limited amount of individual free fibrils immobilized on the mica surface accessible for quantitative characterization. Free fibrils were rather short—the length being approximately a few hundreds of nanometers—but fibrils as long as 1–2 μm were also occasionally observed. AFM-imaged fibrils did not herein show clear longitudinal periodicity although we observed this with TEM (Figures 1, 2). To characterize fibrils their height was measured from AFM images as an indicator of the fibril diameter. Only fibrils longer than 200 nm were included into the height analysis. Hereby we deliberately avoided imaging of oligomers. To acknowledge the fact that the height was not uniform along its whole length, each individual fibril was measured 3 times (close to both ends and in the middle of the fibril) and all three measurements were included in further analysis instead of averaging (to avoid introducing artificial height values). Fibrils that had cohered into bigger aggregates were not accessible for height measurements the same way as the free fibrils. Therefore, fibrils with one end sticking out of the aggregate were defined as entangled and their height was measured once at the free visible part. To investigate whether the free and entangled fibrils belong to the same or different populations of mature fibrils (e.g., one subpopulation with the tendency to aggregate and one without) their height values were collected and analyzed separately.

To visualize the distribution of fibril height a histogram was created for each time point of the fibrillation process for free and entangled fibrils (Figure 5B). AFM image analysis showed fibrils with the average height, i.e., diameter, of 2.5–3.0 nm at 6 h which increased to 5.0–5.5 nm at 13 h of fibrillation (Figure 5B). There were no height-differences between free and entangled fibril populations, demonstrating that the polymorphism is on the level of individual fibrils and not merely fibril stacking.

## Discussion

The question of how normally soluble and functional proteins and peptides are deposited as insoluble amyloid fibrils in the human body attracts much attention in terms of physiological and clinical relevance and importance. Amyloidogenicity and aggregation is a generic property of many proteins and although the resulting protein assemblies share many common features, the deposits both *in vivo* and *in vitro* also display differences that can be monitored at different scales using a wide variety of microscopic and biophysical techniques. While much interest has been focused on initial events in Aβ amyloid fibrillation and oligomerization less interest has been focused on amyloid fibril polymorphism development in the deposited stage. *In vivo* live imaging of different mouse models of AD pathology suggest that formation of novel plaques at old mouse age is rare and that plaques increase in size at a slower rate once they have grown large (Burgold et al., 2011; Hefendehl et al., 2011). Early stages of fibril formation mechanisms and the role of oligomers in pathogenicity have dominated the literature for several years (Lee et al., 2011; Auer et al., 2012). Lately more and more attention has been drawn to the understudied dynamics of morphological rearrangement taking place within amyloid deposits during fibril growth when nascent substrate is being recruited but also at the equilibrium phase of amyloid formation. The clinical need to recognize and understand amyloids, their formation and structural reorganization in relation to the various diseases and their symptoms stimulates studies of amyloid formation and morphology (Hubin et al., 2014) as well as propagation (Eisele and Duyckaerts, 2016). A recent study performed by Kim and co-workers suggests that disaggregation of Aβ plaques in transgenic mice rescue a degenerative disease phenotype (Kim et al., 2015) which implicates that amyloid plaques are indeed involved in disease progression and also underlines the necessity of understanding the molecular structures within the amyloid plaques.

Amyloid fibril structures are mostly studied using techniques involving X-ray fiber diffraction (Serpell et al., 1997; Makin et al., 2005; Marshall et al., 2011), electron microscopy (EM; Jiménez et al., 1999; Zhang et al., 2009; Anderson and Webb, 2011), atomic force microscopy (AFM) (Chamberlain et al., 2000; De Jong et al., 2006; Smith et al., 2006; Adamcik et al., 2010), solid state nuclear magnetic resonance (NMR; Nielsen et al., 2009; Tycko, 2011), and electron paramagnetic resonance (EPR; Török et al., 2002; Sepkhanova et al., 2009), optical and IR spectroscopy (Lindgren and Hammarström, 2010; Middleton et al., 2012). These methods provide detailed information about fibril conformations. However, it is not straight forward to apply them on tissue samples and they cannot be used for imaging of living animals.

We have recently designed a strategy which allows detection of different morphologies of Aβ amyloid derived both *in vitro* and *in vivo* using a mixture of two luminescent conjugated oligothiopenes (LCOs) with different binding propensities and fluorescent signatures (Klingstedt et al., 2011; Nyström et al., 2013). These tools allow us to translate *in vivo* observations to pure simplistic systems *in vitro*. h-FTAA is a heptameric LCO that fluoresces upon binding to assemblies of Aβ1-40 during the early as well as late states of fibrillation with a fluorescence emission maximum at 540 nm whereas q-FTAA is a tetrameric LCO that reports at a later mature stage with a fluorescence emission maximum at 500 nm (Klingstedt et al., 2011).

In this paper we analyzed in detail what dictates the differentiated binding properties of the two LCO probes with amyloids at different maturation stages. The study was dedicated to Aβ1-40 as it induces prominent spectral shifts in the LCOs over time (Nyström et al., 2013). It has also been shown that reorganization is more readily ongoing in Aβ1-40 fibrils than for Aβ1-42 fibrils (Sánchez et al., 2011). Recent studies where LCOs proved to be potential disease modifier drug candidates in prion disease strengthen the urge to achieve detailed understanding of fibril morphology and how it is coupled to ligand binding (Herrmann et al., 2015). By employing combined time scan transmission electron microscopy, atomic force microscopy imaging and fluorescence monitoring of Aβ1-40 during fibrillation we concluded that the maturation phase in the amyloidogenesis of Aβ1-40 *in vitro* includes a phase where solitary fibrils mature into bundles. This step is crucial for formation of a second binding site that enables q-FTAA to bind and fluoresce.

**Figure 6:**
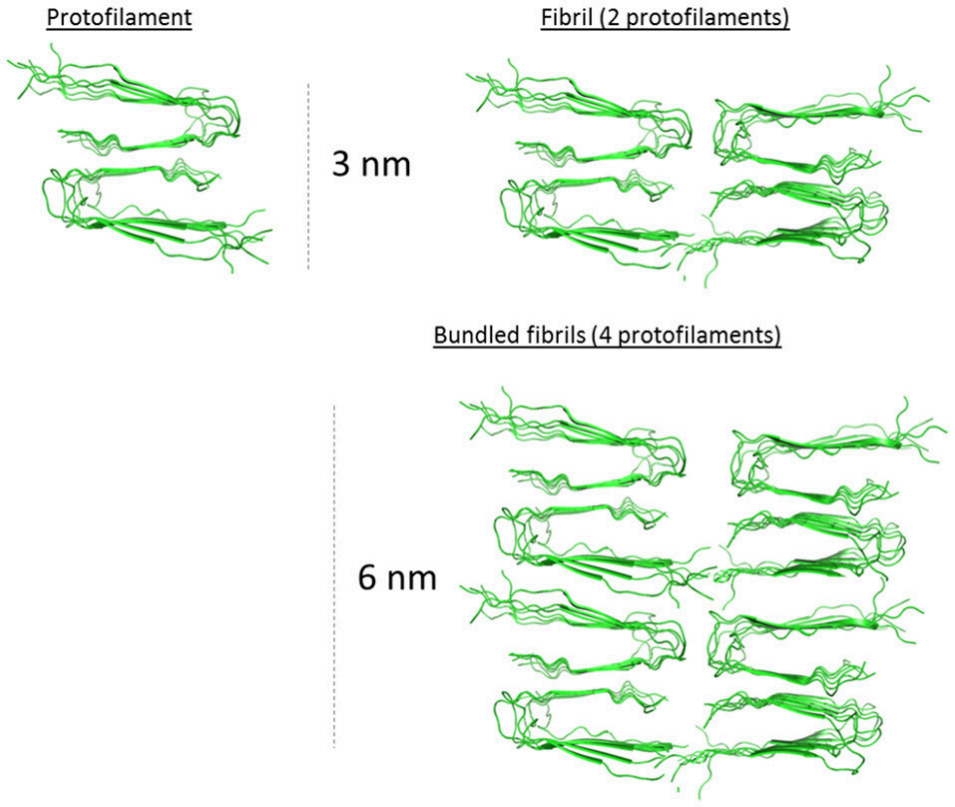
**2.5–3.0 nm protofilament of Aβ1-40 derived from the solid state NMR structure model from Petkova et al. (2006)**. Hypothetical assembled protofilaments for discussions regarding the macro-arrangement of the filaments in the 5.0–5.5 nm bundled fibril as determined by AFM (Figure 5). PDB coordinates from 2 LMO.

The evolution of the fibrils height distribution obtained from the AFM images (Figure 5B) suggested the occurrence of three events within the process of fibrillation: building up individual fibrils (identified as solitary fibrils in TEM images (Figure 5C) into thicker structures (described as bundled fibrils in TEM analysis) resulting in shifting the height distribution toward higher values as well as introduction newly formed fibrils with the lowest height values causing a broadening of the distribution. On top of this, both monofilamentous and bifilamentous fibrils, form large entangled networks as deduced by AFM (Figure 5C). The two main types of analyzed fibrils had average heights, i.e., diameters, of 2.5–3 nm at 6 h up to ~5.0–5.5 nm at 13 h of fibrillation. While this is an oversimplification of the known polymorphism of Aβ1-40 (Tycko, 2014), the dimensions demonstrated by AFM correlates well with the fibrillar structure proposed by the Tycko group (Petkova et al., 2006). Using this prevalent two-fold symmetric Aβ1-40 structural model placing the protofilaments (3 nm high) paired side by side to form 3 nm monofilamentous fibrils with four monomers in the cross-section, and as proposed here to further bundle to form 6 nm wide fibrils (Figure 6).

**Figure 7:**
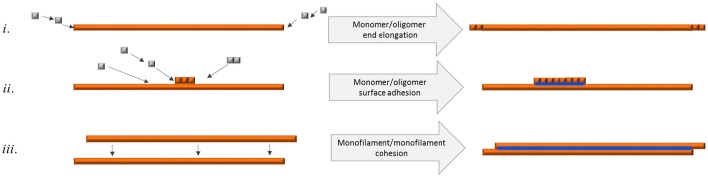
**Schematic figure of different means of fibril elongation and association and how it affects q-FTAA binding propensity**. Orange depicts h-FTAA positive fibrils, blue depicts q-FTAA binding sites **(i)** End addition of monomers or oligomers does not generate the q-FTAA binding site. **(ii)** Monomer or oligomer adhesion to the long end fibril surface generates additional binding site that enables q-FTAA binding. **(iii)** Cohesion of two monofilaments generates the q-FTAA binding site.

Bundled fibrils were often found where the individual fibrils were of equal length displaying blunt ends, implicating that bundled fibrils form through a mechanism of growth along a preformed fibril, metaphorically similar to DNA polymerization (Figure 7ii). It is also possible that a requirement for mature bundled fibrils to accumulate is pairing of single filaments implicating that unpaired filaments break off and eventually find another bundling partner with complementary surface features (Figure 7iii).

Unpaired strands may be unstable and eventually dissolve into oligomers. While we cannot rule out that the observed spherical oligomers are important for fibril maturation, their lack of LCO fluorescence and continuous presence in the soluble fraction does not allow us to directly correlate them with fibrils with these diverse attributes.

Assuming that during the fibril formation, thin monofilamentous fibrils (containing 2 protofilaments) cooperatively assemble with each other leading to the formation of bundled multi-filamentous fibrils, this increase in fibril height can be the result of more and more individual fibrils being combined together into mature and stable structures. At the next level of complexity, the individual fibrils, thin solitary ones as well as thicker bundled entities become entangled forming spacious 3-dimensional networks. However, the entanglement of fibrils was not coupled to the binding propensity of q-FTAA. On the contrary, monofilamentous and bifilamentous fibrils (by AFM) were equivalently present in free and entangled fibrils (Figures 5B,C). The sample condition in our studies (10 μM Aβ1-40, 37°C, pH 7.4) was almost identical previous experiments (Lee et al., 2011) which corresponds to conditions where nucleated conformational conversion and nucleated polymerization both occur (Auer et al., 2012). As expected from these previous studies, spherical oligomers were detected both by AFM and TEM. In our studies we did not observe a dominant presence of fibril-end-associated spherical oligomers, but these were rather associated randomly with- and away from fibrils and oligomers were also omnipresent at all time points both by TEM and AFM imaging. However, the lack of LCO fluorescence from oligomers in our study rendered us unable to include these species in the interpretation of the data. This does not rule out these as potentially important species for fibril formation and maturation but our read out is confined to the presence of fibrils simply defined as two main types (single and bundled).

The presence of both entangled and free fibrils appeared over the entire time scan imaging but a specific increase in fibril diameter over time, in concordance with increase in bundling as deduced by TEM strongly promotes the hypothesis that it is the assembly of single filament fibrils into intertwined, thicker multi-filamentous fibrils that generates a new binding site that allows q-FTAA to adhere in a fluorescent mode (Figure 7). When spherical oligomers separated from fibrils via ultracentrifugation were monitored by LCO fluorescence they failed to display a fluorescence profile that can explain the early-stage (< 10 h) fluorescence signal preceding the q-FTAA fluorescence (>10 h; Figure 2). This finding supports that h-FTAA fluorescence at early time points is predominantly exhibited from LCOs bound to single-filament fibrils.

The conformational rearrangement herein described *in vitro* is detectable in Aβ plaques *in vivo* in transgenic mice when monitored by conformation-sensitive oligothiophenes (Nyström et al., 2013). This rearrangement is however not uniform throughout the amyloid plaque. As the mice age, the plaque cores displayed increasing q-FTAA fluorescence while the periphery of the plaques remained essentially refractive to q-FTAA but is readily stained by h-FTAA. APP23 mice that display a high Aβ1-40/Aβ1-42 ratio (Sturchler-Pierrat et al., 1997) serve as a good model for exploring the fibrillary arrangement in closer detail by using conformation sensitive dyes in combination with the monoclonal Aβ antibody 4G8. Monitoring the staining topology of a plaque formed *in vivo*, there was a close relationship between increase in q-FTAA fluorescence and decrease in antibody signal from triple stained APP23 mice. This staining topology supports that increase in q-FTAA binding in the plaque core also *in vivo* is coupled to decrease in the number of available 4G8 epitopes.

The feature of increased number of hidden epitopes can be back tracked to *in vitro* derived Aβ1-40 fibrils using an SPR based approach where Aβ fibrils are simultaneously injected over channels that have been coated with q-FTAA and h-FTAA, respectively. A subsequent injection of the monoclonal Aβ antibody 4G8 reveals that more antibody epitopes are available on fibrils that have been bound to h-FTAA than on those that have been captured by q-FTAA, again suggesting that increased affinity between Aβ fibrils and q-FTAA is associated with decrease in antibody epitopes which, in turn, corroborates fibril bundles as a key requirement for q-FTAA binding both *in vitro* and *in vivo*.

As we have shown in this and previous studies morphologic amyloid fibril rearrangement during maturation is an important but understudied process. Awareness of this process and its relation to fibril polymorphism is important and studies of these processes are crucial for further understanding of the disease process and for design of new therapeutic interventions and diagnostic tools. Addressing fibril polymorphism at the molecular level *in vivo* in living organisms and in autopsy or biopsy tissue samples using high resolution spectroscopy and microscopy techniques is not readily executed. With the opportunities provided by conformation sensitive probes we can monitor fibril maturation *in vitro*. The unique possibility offered by combining fluorescence results with other biophysical measurements provides detailed understanding of the dynamics of Aβ amyloid structures. This can be translated back to the *in vivo* derived amyloid depositions by LCO fluorescence readout and help us understand how different fibril morphologies influences disease progression. This, in turn, will provide more knowledge of how to understand and tackle Alzheimer's disease.

## Author contributions

KP designed and performed research, provided analytic tools, analyzed data, wrote the manuscript. PH designed and performed research, analyzed data. LJ designed and performed research, analyzed data. ML contributed analytical tools and strategies and analyzed data. BS provided analytic tools and strategies, analyzed data, and wrote the manuscript. KN provided analytic tools and strategies, analyzed data. SN designed and performed research, analyzed data, and wrote the manuscript.

## Funding

We wish to acknowledge the following financial support: FUGE project supported by the Norwegian Research Council contract 183338/S10 (KP and BS), The Swedish Alzheimer Foundation AF-555511 (SN), Göran Gustafsson Foundation (PH), Swedish Research Council (PH), Swedish Research Council 2015-04521 (PH), The Swedish Brain Foundation (PH), and the Swedish Foundation for Strategic Research (KN). ML is grateful to Linköping University for a guest professor tenure during which a part of this work was carried out.

### Conflict of interest statement

SN, PH, and KN are minor shareholders in Ebba Biotech which commercializes hFTAA under the brand name AmyTracker545. KP, LJ, ML, and BS declare that the research was conducted in the absence of any commercial or financial relationships that could be construed as a potential conflict of interest.
